# Which dietary patterns fend off nonalcoholic fatty liver disease? A systematic review of observational and interventional studies

**DOI:** 10.1186/s40795-024-00961-8

**Published:** 2024-11-28

**Authors:** Farnush Bakhshimoghaddam, Daniel Baez, Neda Dolatkhah, Mahdi Sheikh, Hossein Poustchi, Azita Hekmatdoost, Stanford Dawsey, Farin Kamangar, Christian Abnet, Reza Malekzadeh, Arash Etemadi, Maryam Hashemian

**Affiliations:** 1 Nutrition and Metabolic Diseases Research Center, Clinical Sciences Research Institute, Ahvaz Jundishapur University of Medical Sciences, Ahvaz, Iran; 2 Department of Nutrition, School of Allied Medical Sciences, Ahvaz Jundishapur University of Medical Sciences, Ahvaz, Iran; 3 Department of Biology, School of Arts and Sciences, Utica University, Utica, NY USA; 4 Physical Medicine and Rehabilitation Research Center, Tabriz University of Medical Sciences, Tabriz, Iran; 5 Genomic Epidemiology Branch, International Agency for Research on Cancer (IARC/WHO), Lyon, France; 6 Liver and Pancreatobiliary Diseases Research Center, Digestive Diseases Research Institute, Tehran University of Medical Sciences, Tehran, Iran; 7 Departments of Clinical Nutrition and Dietetics, Faculty of Nutrition and Food Technology, National Nutrition and Food Technology Research Institute, Shahid Beheshti University of Medical Sciences, Tehran, Iran; 8 Metabolic Epidemiology Branch, Division of Cancer Epidemiology and Genetics, National Cancer Institute, National Institutes of Health, Bethesda, MD USA; 9 Digestive Oncology Research Center, Digestive Diseases Research Institute, Tehran University of Medical Sciences, Tehran, Iran; 10 Department of Biology, School of Computer, Mathematical, and Natural Sciences, Morgan State University, Baltimore, MD USA; 11 Epidemiology and Community Health Branch, Division of Intramural Research, Blood Institute, National Heart, National Institutes of Health, Lung, Bethesda, MD USA

**Keywords:** Dietary patterns, Hepatic steatosis, Nonalcoholic fatty liver disease, Nonalcoholic steatohepatitis, Mediterranean diet, DASH, MAFLD

## Abstract

**Background:**

The global burden of non-alcoholic fatty liver disease (NAFLD) has significantly risen over the past decade. Dietary intake strongly influences its development and should be a component of any prevention and treatment plan strategy. Dietary pattern analysis enables the investigation of the overall diet and permits the consideration of interactions and cumulative effects of dietary components. The current study aimed to systematically review observational studies and intervention trials to determine the associations between various dietary patterns and NAFLD.

**Methods:**

The protocol was written according to the Preferred Reporting Items for Systematic Reviews and Meta-Analyses (PRISMA) guidelines. We searched PubMed, Embase, and the Cochrane Library. We included studies that reported a priori dietary pattern (i.e., diet quality scores) or *a posteriori* method, which identified existing eating patterns (i.e., principal component analysis) in adult participants. Two investigators conducted independent screening, extraction, and quality assessment using the Newcastle‒Ottawa or Jadad scale. A third reviewer resolved conflicts.

**Results:**

We identified 27 relevant observational and 16 interventional studies from 16 countries. A Mediterranean or DASH diet might prevent and improve NAFLD, whereas dietary patterns such as Western dietary patterns characterized by high consumption of sweets and animal foods such as red meat and fast food are positively associated with NAFLD. A low-carbohydrate diet effectively prevents and treats NAFLD; however, we need more research on the effects of a low-fat diet and the type of fats.

**Conclusion:**

Healthy dietary patterns, mainly plant-based or adjusted macronutrient distributions, such as the adoption of a low-carbohydrate diet, are linked to a reduced risk of NAFLD and could halt its progression. We proposed recommendations for future studies to fill the gap in knowledge regarding the management of NAFLD via dietary modifications.

## Introduction

Nonalcoholic fatty liver disease (NAFLD) is defined as a hepatic triglyceride content of more than 5% in the absence of significant alcohol consumption or any secondary causes for hepatic steatosis [1]. NAFLD encompasses a broad spectrum of liver dysfunctions, ranging from simple steatosis to nonalcoholic steatohepatitis (NASH) and cirrhosis that may progress to hepatocellular carcinoma [2]. As NAFLD is linked to an increased risk for metabolic disease conditions, the prevention and management of NAFLD is one of the major public health challenges [1].

There is no specific treatment for NAFLD. However, lifestyle interventions, including physical activity, weight reduction, and dietary modification, are recommended as the primary options for the therapeutic management of NAFLD [3]. Since single nutrients or food groups are not consumed alone, exploring overall diet as a dietary pattern has been suggested to provide a more comprehensive view of the relationship between diet and chronic diseases, especially as components of these patterns may interact antagonistically or synergistically [4].

Despite considerable research, dietary strategies for the nutritional management of NAFLD are still an open issue. The optimal distribution of macronutrients to improve NAFLD is unclear. Recent studies reported that improving adherence to healthy dietary patterns, including the Mediterranean diet (MD) and Dietary Approaches to Stop Hypertension diet (DASH), which are characterized by an abundance of fruits, vegetables, and whole grains, may have an inverse association with hepatic steatosis [5]. Two systematic reviews of observational studies suggested that an unhealthy dietary pattern (a high intake of high-fat dairy products, red and processed meats, refined grains, and sweets) is associated with an increased risk of NAFLD. Conversely, a healthy diet (high in whole grains, legumes, fruits, vegetables, poultry, and fish) was associated with a reduced risk for NAFLD [6, 7]. However, interventional studies provide better evidence of any effects. A systematic review of only three interventional studies evaluating fatty liver by biopsy, MRI, and MRS demonstrated that MD reduced hepatic fat content [8]. Therefore, in this systematic review, we reviewed all studies that diagnosed and evaluated fatty liver disease with common NAFLD assessment tools and included both observational and interventional studies in this review to systematically review all available evidence and to ascertain the associations between dietary patterns consumed, macronutrient distribution and NAFLD.

## Methods

The protocol for our systematic review was written using the Preferred Reporting Items for Systematic Reviews and Meta-Analyses (PRISMA) statement guidelines [9]. The purpose of the study was to determine the relationship between dietary patterns and NAFLD, and the protocol was registered in the International Prospective Register of Systematic Reviews (PROSPERO) database (CRD42022340506). We included randomized and nonrandomized interventions and observational studies that evaluated dietary patterns and NAFLD in adult participants.

### Search strategy

A comprehensive search was conducted in PubMed, Embase, and the Cochrane Central Register of Controlled Trials. The merger of MeSH and non-MESH terms were composed by experts in the field and included the following terms: (“diet” OR “food” OR “eating” OR “eat” OR “dietary” OR “feeding” OR “nutrition” OR “nutrient” OR “dietary score” OR “Diet Quality Index” OR “Food Score” OR “Diet Score” OR “MedDietScore” OR “Dietary Pattern Score” OR “healthy eating index” OR “diet quality” OR “dietary pattern” OR “diet pattern” OR “eating pattern” OR “food pattern” OR “eating habit” OR “dietary habit” OR “food habit” OR “dietary profile” OR “food profile” OR “diet profile” OR “eating profile” OR “dietary guideline” OR “dietary recommendation” OR “food intake pattern” OR “dietary intake pattern” OR “diet pattern” OR “eating style” OR “DASH” OR “dietary approaches to stop hypertension” OR “Diet, Mediterranean” OR “Mediterranean” OR “vegan” OR “vegetarian” OR “Diet, Vegetarian” OR “prudent diet” OR “Western diet” OR “southern diet” OR “omniheart” OR “Optimal Macronutrient Intake Trial to Prevent Heart Disease” OR “Okinawa” OR “Ethnic Groups” OR “plant based”) and (“Non-alcoholic fatty liver disease” OR “Non alcoholic fatty liver disease” OR “NAFLD” OR “Non-alcoholic steatohepatitis” OR “Non alcoholic steatohepatitis” OR “NASH” OR “MAFLD” OR “Metabolic Fatty Liver Disease” OR “MASLD” OR “Metabolic Dysfunction-Associated Steatotic Liver Disease”). To prevent missing relevant studies, a manual search of reference lists cited was conducted to identify articles that may not have been included within the electronic databases searched.

### Eligibility criteria

Studies with the following conditions were included in this systematic review: (a) cohort, case-control, cross-sectional studies, and clinical trials; (b) performed on individuals over 18 years of age; (c) reported a priori dietary patterns (hypothesis-driven approach) or *a posteriori* dietary patterns (exploratory approach); and (d) diagnosed NAFLD using valid methods, including liver biopsy, imaging methods or validated scores that predict NAFLD using biomarkers. The studies were restricted to those published in English up until September 2021. Table 1 shows the inclusion and exclusion criteria in detail.

**Table 1:** The inclusion and exclusion criteria for study selection

Category	Inclusion criteria	Exclusion criteria
Study design	Cohort Studies Case-control Studies Cross-sectional Studies Randomized clinical trials	Animal Studies Cellular and Molecular Studies Systematic reviews
Intervention/Exposure	Studies that examined consumption of and/or adherence to a dietary pattern, dietary score, or dietary index (i.e., Mediterranean Diet, Healthy Eating Index)	Did not provide a description of the dietary pattern Examined consumption of a single macronutrient vs. patient outcome
Comparison	Individuals in highest category of dietary scores compared to those in lowest category	NA
Outcomes	Nonalcoholic fatty liver disease	Alcoholic fatty liver disease
Language of publication	English	Language other than English
Study participants	Human participants Male participants Female participants	Non-human participants
Age of study participants	Adults (aged 18–64 y) Older adults (aged 65 y or older)	Children and adolescents (aged 2–17 y)
Health status of study participants	Studies that enrolled participants who were healthy and/or at risk for NAFLD Studies that enrolled participants who were diagnosed with NAFLD	Studies that enrolled participants who were diagnosed with a disease or injury not related to NAFLD
Date of publication	No limit to September 2021	NA

### Data extraction

Two independent reviewers (FB and DB) screened the titles and abstracts of the relevant studies and conducted the study selection, while a chief investigator (MH) was also present to resolve any disagreements. The full texts of potentially eligible articles were reviewed to identify relevant studies for inclusion. Two dietary pattern approaches were used: a priori methods, using dietary indices to evaluate the adherence of participants to a priori defined dietary patterns, such as the MD or *a posteriori* methods, using factor analysis, cluster analysis, or reduced rank regression to aggregate the participants into groups such as the Western diet. The following data were recorded from each study: first author’s name, year of publication, method of diagnosing NAFLD, study design and duration, study location, study characteristics/group, dietary assessment tool, dietary pattern approach (*a posteriori* or a priori), type of dietary pattern (MD, and other), corresponding odds ratio (OR), relative risk (RR), or hazard ratio (HR) with 95% CI, and *P* value. We extracted the OR, RR, or HR values with the most adjustment models.

These two investigators also independently assessed the quality of each study by utilizing the Newcastle‒Ottawa scale [10] for cohort, case-control, and cross-sectional studies and utilizing the Jadad scale [11] for randomized controlled trials. The Newcastle‒Ottawa scale assigns up to a maximum of nine points for the least risk of bias in three domains: (a) selection of study groups (four points); (b) comparability of groups (two points); and (c) determining exposure and outcomes (three points) for case-control and cohort studies, respectively [10]. The Jadad scale utilizes a 3-item scale covering the randomization method, the blinding method, and withdrawals/dropouts. The Jadad scores range from 0 to 5, with ≥ 3 points indicating a high-quality study and ≤ 2 points indicating a low-quality study [11]. The chief investigator resolved any discrepancies in the scores given to each paper.

## Results

### Description of studies

**Fig. 1:**
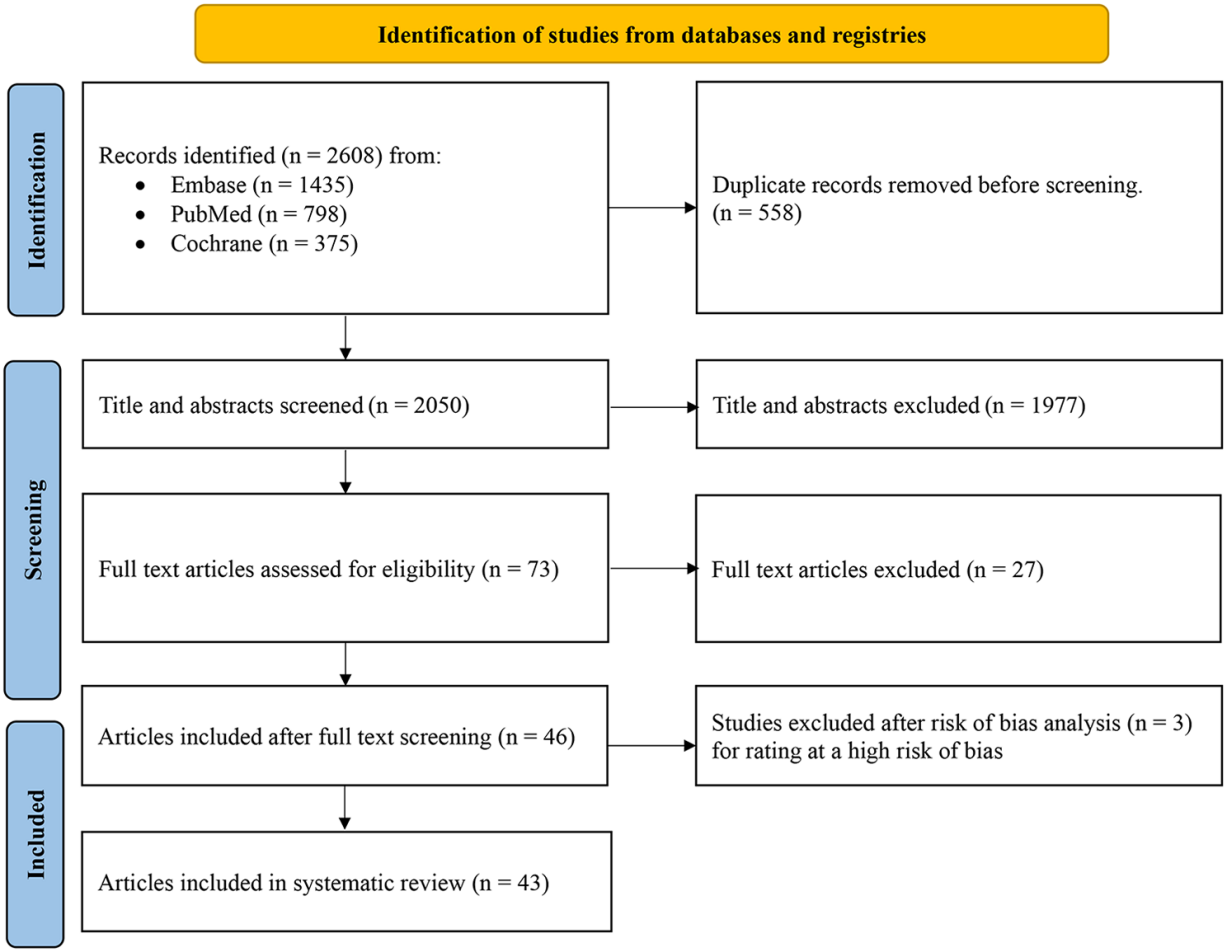
PRISMA flowchart of the search results and the included studies

After searching the databases, we identified 2608 references, which were reduced to 2050 after excluding deduplications. Of the 2050 references screened by title and abstract, 73 went forward for inclusion and exclusion by reading the full text. Most studies had a low or moderate risk of bias, except for two studies, which scored below two according to the Jedad score. These were not included in the analysis [12, 13]. Additionally, we excluded one study as it had been flagged with an expression of concern by the publishers [14]. The body of evidence included 43 articles, 27 of which were observational studies [5, 15–40] and 16 of which were interventional trials [41–56] (Fig. 1).

**Fig. 2:**
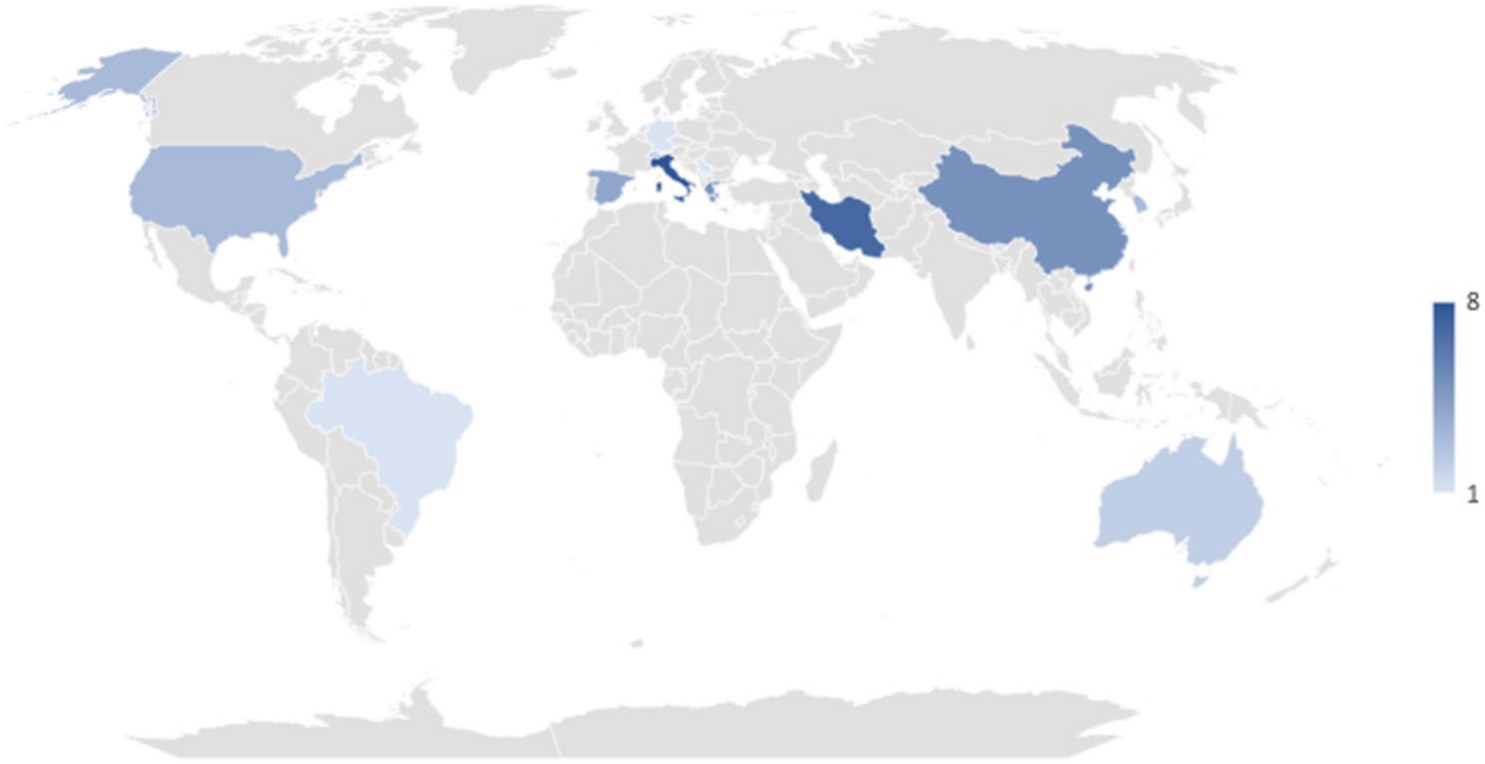
Distribution of countries included in the systematic review

Most studies included both males and females in their interventions (between 10 and 60% females), and only two studies were limited to males [52, 55]. The studies were conducted in 16 different countries. A total of 19 studies originated from Europe [5, 16, 17, 23, 27–31, 41–44, 46, 49, 51–53, 55], 17 from Asia and the Middle East [18–22, 24–26, 34–40, 47, 50], and six from North America and Australia [15, 32, 33, 45, 54, 56] (Fig. 2).

Dietary variables were measured by using a variety of instruments. Most studies utilized validated FFQs [5, 16–18, 20–23, 25–35, 37–41, 46–50, 53, 55, 57], whereas the remaining studies used dietary recalls (*n* = 1) [15], food records (*n* = 5) [24, 36, 45, 51, 56], a combination of dietary assessment tools (*n* = 1) [44], a modified Burke diet history interview (*n* = 1) [54], and not specified (*n* = 4) [19, 42, 43, 52].

NAFLD was assessed using liver biopsy [30, 44]; imaging methods, including ultrasound [15, 17, 19–24, 26–29, 34–39, 41–43, 48, 51, 52, 55, 56], transient elastography [16, 25, 30, 46], CT scan [32, 40], magnetic resonance imaging (MRI) ) [5, 47, 49, 57], magnetic resonance spectroscopy (MRS) [18, 45, 50, 54], and nuclear magnetic resonance (NMR) [53]; or scores predicting NAFLD severity, including the fatty liver index (FLI) or triglyceride-glucose (TyG) criteria [28, 29, 31], and NAFLD score [28, 29]. The FLI includes several criteria, such as body mass index (BMI), waist circumference, gamma-glutamyl transferase (GGT), and triglyceride levels. TyG includes triglycerides and fasting plasma glucose. The NAFLD score includes the presence of metabolic syndrome, type 2 diabetes, fasting concentrations of insulin, aspartate aminotransferase (AST), and the aspartate aminotransferase (AST)/alanine transaminase (ALT) ratio [28, 29].

The results of the studies were reported according to study design, observational (Table 2) or interventional studies (Table 3).

**Table 2:** Characteristics of observational studies investigating the association of dietary patterns with nonalcoholic fatty liver disease

Author, Year (Reference)	Study Design	Study Characteristics	Method of NAFLD diagnosis	Dietary assessment tool	Dietary pattern	Method of Analysis	Effect Estimate (95%CI)	Quality assessment
*Priori* Dietary Patterns								
Park et al. 2020 [33]	Nested case-control within the Cohort	*n* = 32,251 (2959 with NAFLD and 29,292 without) in the Multiethnic Cohort study (62% female) age 45–75 USA	linkage to 1999–2016 Medicare claims	FFQ	aMDS	OR Q5 vs. Q1	1.0 (0.88–1.15)	8
					AHEI		0.91 (0.8–1.03)	
					HEI		0.83 (0.73–0.94)	
					DASH		0.78 (0.69–0.89)	
Ma et al. 2018 [32]	Cohort	*n* = 1521 participants with liver fat measured at Framingham Heart Study median follow-up: 6 y (51% female) mean age: 57 ± 10 USA	CT scan	FFQ	Δ MDS	OR Q4 vs. Q1	Liver fat decreased: 0.57 (0.27–0.86) Incident fatty liver decreased: 26% (95% CI, 10-39%)	8
					Δ AHEI scores		Liver fat decreased: 0.56 (0.29–0.84) Incident fatty liver decreased: 21% (5-35%)	
Kouvari et al. 2021 [31]	Nested case-control within the Cohort	*n* = 3042 (1263 with NAFLD) in the ATTICA study follow-up: 10 y (51% female) Mean age: men: 46 ± 13 and women: 45 ± 14 Greece	TyG index	FFQ	MDS	OR T3 vs. T1	0.53 (0.29–0.95)	7
Alferink et al. 2020 [16]	Cohort	*n* = 343 patients with NAFLD in the Rotterdam Study median follow-up: 4.4 y (56% female) age 69–73 Netherlands	Transient elastography	FFQ	MDS	OR per 1-unit increase in pattern score	0.84 (0.66–1.05)	7
					Dutch Dietary Guidelines (healthy)		0.89 (0.71–1.12)	
					WHO-score		0.73 (0.53-1.00)	
Khalatbari-Soltani et al. 2020 [29]	Cohort	*n* = 153 patients with NAFLD (FLI) in the Swiss CoLaus Study follow-up: 5.3 y (65.4% female) mean age: 55.8 ± 10.0 Switzerland	FLI criteria and NAFLD score	FFQ	MDS	RR Q5 vs. Q1	FLI: 0.85 (0.71–1.02) NAFLD score: 0.95 (0.83–1.09)	8
Giraldi et al. 2020 [23]	Case-control	*n* = 815 (371 with NAFLD and 444 without) (37% female) mean age: case: 59 ± 16.0 and control: 45 ± 14.4 Italy	Ultrasound	FFQ	MDS	OR per 1-unit increase in pattern score	0.83 (0.71–0.98)	7
Watzinger et al. 2020 [5]	Cross-sectional	*n* = 136 patients with NAFLD (50.7% female) age 35–65 Germany	MRI	FFQ	MDS	OR Q1 vs. Q4	4.41 (1.28–15.15)	4
					DASH score		4.41 (1.44–13.48)	
Baratta et al. 2017 [17]	Cross-sectional	*n* = 584 patients with NAFLD (38.2% female) mean age: 55.8 ± 10.0 Italy	Ultrasound	FFQ	MDS	OR per 1-unit increase in pattern score	0.090 (0.01–0.79)	5
Khalatbari-Soltani et al. 2019 [28]	Cross-sectional	*n* = 2305 patients in the Fenland cohort and 1001 patients in the CoLaus cohort study, England and Switzerland	Ultrasound, FLI criteria, and NAFLD score	FFQ	MDS	PR Q5 vs. Q1	FLI: 0.82 (0.78–0.86) in Fenland Study FLI: 0.85 (0.80–0.91) in CoLaus Study	5
Hekmatdoost et al. 2016 [25]	Case-control	*n* = 306 (102 with NAFLD and 204 without) (57% female) mean age: 54.42 Iran	Fibroscan	FFQ	DASH	OR Q4 vs. Q1	0.92 (0.73–1.12)	7
Xiao et al. 2020 [38]	Cross-sectional	*n* = 3051 patients with NAFLD (68% female) age 40–75 China	Ultrasound	FFQ	DASH score	OR Q5 vs. Q1	0.77 (0.61–0.97)	5
Hashemi Kani et al. 2013 [24]	Case-control	*n* = 200 (100 with NAFLD and 100 without) (60% female) mean age: 37.9 Iran	Ultrasound	3-day dietary record	HEI	OR Q1 vs. Q4	1.88 (0.76–2.95)	6
					Dietary diversity score		1.76 (0.68–2.89)	
					Dietary energy density		0.53 (0.19–0.89)	
					Mean adequacy ratio of nutrients		2.03 (0.91–4.03)	
*Posteriori* Dietary Patterns								
Salehi-sahlabadi et al. 2021 [34]	Case-control	*n* = 675 (225 with NAFLD and 450 without) (47% female) mean age: control: 37.88 ± 8.92 and case: 38.63 ± 8.71 Iran	Ultrasound	FFQ	WDP	OR T3 vs. T1	3.64 (2.52–5.32)	8
					HDP		0.30 (0.13–0.68)	
					TDP		1.21 (0.57–2.57)	
Tutunchi et al. 2021 [36]	Case-control	*n* = 210 (105 with NAFLD and 105 without) (57.2% female) age 30–60 Iran	Ultrasound	3-day dietary record	VLFD (healthy)	OR T3 vs. T1	0.33 (0.11–0.84)	8
					SHMS (unhealthy)		3.17 (1.04–6.66)	
Xia et al. 2020 [37]	Case-control	*n* = 4086 (2043 with NAFLD and 2043 without) based on TCLSIHealth Cohort Study (42% female) China	Ultrasound	FFQ	Sweet pattern	OR Q4 vs. Q1	1.01 (0.84–1.22)	8
					AFP		1.23 (1.03–1.48)	
					TDP		0.92 (0.77–1.10)	
Dehghanseresht et al. 2020 [21]	Case-control	*n* = 244 (122 with NAFLD and 122 without) (56% female) age 19–70 Iran	Ultrasound	FFQ	FFP	OR T3 vs. T1	0.72 (0.26–1.96)	7
					TDP		3.58 (1.48–8.68)	
					Vegetables and Dairy		0.23 (0.09–0.58)	
					Ordinary pattern		3.74 (1.23–11.42)	
Kalafati et al. 2019 [27]	Case-control	*n* = 351 (134 with NAFLD and 217 without) (57% female) mean age: 50.4 Greece	Ultrasound	FFQ	Unsaturated fatty acids	OR Q4 vs. Q1	0.82 (0.36–1.85)	7
					Prudent		0.70 (0.30–1.62)	
					High protein pattern		1.67 (0.71–3.93)	
					FFP		4.24 (1.58–11.32)	
Fakhoury-Sayegh et al. 2017 [22]	Case-control	*n* = 222 (112 with NAFLD and 110 without) (55% female) the mean age for cases: 39.9 ± 6.0 and for control: 38.8 ± 13.2 Lebanese	Ultrasound	FFQ	High-meat fast food	OR per 1-unit increase in pattern score	4.08 (1.35–12.28)	8
					High fruit		4.06 (1.32–12.10)	
					TDP		0.30 (0.10–0.85)	
Chung et al. 2019 [20]	Cross-sectional	*n* = 331 patients with NAFLD (35.9% female) mean age: 51.1 South Korea	Ultrasound	FFQ	TDP	OR Q5 vs. Q1	1.85 (1.11–3.08)	5
					Western and high-carbohydrate		1.58 (0.92–2.73)	
					Simple meal pattern		0.59 (0.34-1.00)	
Ghaemi et al. 2018 [40]	Cross-sectional	*n* = 1500 patients with NAFLD (42% female) mean age: 34.9 Iran	CT scan	FFQ	Healthy	OR Q4 vs. Q1	0.61 (0.35–1.07)	4
					Unhealthy		2.73 (1.21–6.15)	
Adriano et al. 2017 [15]	Cross-sectional	*n* = 229 older adults with NAFLD (74.4% female) mean age: 68.7 ± 6.4 Brazil	Ultrasound	24-h Dietary recall	TDP	PR T3 vs. T1	1.05 (0.73–1.52)	4
					Regional snacks		1.42 (1.02–1.92)	
					Energy-dense		1.05 (0.71–1.53)	
					Healthy		0.70 (0.50–0.98)	
Shim et al. 2017 [35]	Cross-sectional	*n* = 58 patients with NAFLD (43.1% female) mean age: 49.32 ± 13.84 South Korea	Ultrasound	FFQ	WDP	OR T3 vs. T1	2.99 (1.08–8.22)	4
					Prudent		0.35 (0.13–0.93)	
Q Jia et al. 2015 [26]	Cross-sectional	*n* = 4206 patients with NAFLD (30% female) mean age: 42 China	Ultrasound	FFQ	Balanced dietary pattern	OR Q4 vs. Q1	Males: 0.80 (0.58–1.10) Females: 0.60 (0.36–1.01)	4
					High protein/ cholesterol		Males: 1.31 (0.98–1.75) Females: 1.04 (0.67–1.61)	
					High carbohydrate/sweet		Males: 0.99 (0.73–1.33) Females: 2.19 (1.40–3.46)	
Yang et al. 2015 [39]	Cross-sectional	*n* = 999 patients with NAFLD (47% female) age: 45–60 China	Ultrasound	FFQ	TDP	PR Q4 vs. Q1	0.83 (0.66–1.06)	4
					AFP		1.25 (0.99–1.59)	
					Grains-Vegetable		0.77 (0.62–0.98)	
					High-Salt		0.91 (0.71–1.17)	
Chan et al. 2015 [18]	Cross-sectional	*n* = 797 patients with NAFLD (58% female) age: 19–72 China	MRS	FFQ	MDS	OR per 1-unit increase in pattern score	0.90 (0.79–1.02)	4
					Diet Quality Index-International		0.79 (0.64–0.97)	
Choi et al. 2015 [19]	Cross-sectional	*n* = 615 patients with NAFLD (38.8% female) mean age: 48.3 South Korea	Ultrasound	-	Vegetarian	OR per 1-unit increase in pattern score	1.27 (0.99–1.64)	4
Kontogianni et al. 2014 [30]	Cross-sectional	*n* = 73 patients with NAFLD (31.5% female) mean age: 45.4 ± 11.3 Greece	Transient elastography and liver biopsies	FFQ	MDS	OR per 1-unit increase in pattern score	1.03 (0.93–1.15)	5

AFP: animal foods pattern; AHEI: alternative healthy eating index; aMDS: alternate Mediterranean diet score; DASH: dietary approaches to stop hypertension; FFP: fast food pattern; FFQ: food frequency questionnaire; FLI: fatty liver index; HEI: healthy eating index; HDP: healthy dietary pattern; MD: Mediterranean diet; MDS: Mediterranean diet score; MRI: magnetic resonance imaging; OR: odd ratio; PR: prevalence ratios; RR: hazard ratio; SHMS: sweet, hydrogenated fat, red and processed meat, and soft drink; TDP: traditional dietary pattern; TCLSIHealth: Tianjin Chronic Low-grade Systemic Inflammation and Health; TyG: Triglycerides-glucose index; VLFD: vegetables, legumes, fruits, and low-fat dairy products; WDP: western dietary pattern

**Table 3:** Characteristics of interventional studies investigating the association of dietary patterns with nonalcoholic fatty liver disease^1^

Author, Year (Reference)	Method of NAFLD diagnosis	Study Design Duration	Study Characteristics	Dietary assessment tool	Study groups	Dietary pattern	Results *P* values (before/after)	*P* values between groups	Quality assessment
Mediterranean Diet (MD)									
Yaskolka Meir et al. 2021 [50]	MRS	RCT, Parallel 18 months	*n* = 294 obesity/dyslipidemia patients with NAFLD (12% female) mean age: 51 Israel	FFQ	PA and energy-restricted diets for all groups (1200–1400 kcal/d for women and 1500–1800 kcal/d for men) 1. HDG (standard nutritional counseling) 2. MD (28 g/day walnuts) 3. green-MD [28 g/day walnuts + 3–4 cups/day green tea + 100 g/d *Wolffia globose* (Mankai strain)]	HDG	Δ IHF%: -12.2%	green-MD vs. MD (*P* = 0.03) green-MD vs. HDG (*P* < 0.001)	5
						MD	Δ IHF%: -19.6%		
						Green-MD	Δ IHF%: -38.9%		
Abenavoli et al. 2017 [42]	Ultrasound	RCT, Parallel 6 months	*n* = 50 overweight patients with NAFLD (40% female) age 40–60 Italy	Not specified	Energy-restricted diets for all groups (1400–1600 kcal/d) with CHO 50–60%, proteins 15–20%, 20–35% fat 1. MD 2. MD + antioxidant complex supplement (silymarin 120 mg, chlorogenic acid 7.5 mg, protopine 0.04 mg, L-methionine 150 mg, L-glutathione 10 mg) 3. Control group (habitual diet)	MD	Δ FLI: -26 ± 13.6* (*P* = 0.002) Δ ALT: 3 ± 6.6* (*P* = 0.49) Δ AST: 1 ± 2.2* (*P* = 0.10) Δ GGT: 5 ± 5.3* (*P* = 0.02)	Δ FLI: *P* = 0.02 Δ ALT: *P* > 0.05 Δ AST: *P* > 0.05 Δ GGT: *P* > 0.05	5
						Control group	Δ FLI: 2.0 ± 7.6* (*P* = 0.07) Δ ALT: 5.0 ± 16.0* (*P* = 0.88) Δ AST: 4.0 ± 17.4* (*P* = 0.02) Δ GGT: 8.0 ± 9.3* (*P* = 0.04)		
Abenavoli et al. 2015 [43]	Ultrasound	RCT, Parallel 6 months	*n* = 30 overweight patients with NAFLD (30% female) age 45–60 Italy	Not specified	Energy-restricted diets for all groups (1400–1600 kcal/day) with CHO 50–60%, proteins 15–20%, 20–35% fat 1. MD 2. MD plus antioxidant complex supplement (silybin 94 mg, phosphatidylcholine 194 mg, vitamin E acetate 89.28 mg) 3. Control group (habitual diet)	MD	Δ FLI: -32 ± 14.2* (*P* = 0.02) Δ ALT: -17 ± 21* (*P* = 0.31) Δ AST: 1 ± 3.1* (*P* = 0.32) Δ GGT: 5 ± 6.3* (*P* = 0.31)	Δ FLI: *P* = 0.009 Δ ALT: *P* > 0.05 Δ AST: *P* > 0.05 Δ GGT: *P* > 0.05	3
						Control group	Δ FLI: 2 ± 7.5* (*P* = 0.11) Δ ALT: 11 ± 17.75* (*P* = 0.68) Δ AST: 4 ± 21.93* (*P* = 0.03) Δ GGT: 10.0 ± 9.93* (*P* = 0.047)		
Katsagoni et al. 2018 [48]	Ultrasound	RCT, Parallel 6 months	*n* = 63 overweight/obese patients with NAFLD (32% female) mean age: 50 ± 11 Greece	FFQ	Energy-restricted diets for all groups (1500 kcal/d for women 1800 kcal/d for men) with 45% CHO, 20% protein and 35% lipids 1. MD 2. Control group (healthy diet lifestyle) 3. MLG (increasing PA and improving sleep habits)	MD	Δ Liver stiffness: -0.4 ± 2.1 (*P* < 0.05) Δ NAFLD fibrosis score: -2.48 ± 1.5 (*P* > 0.05) Δ ALT: -17 ± 21* (*P* > 0.05) Δ GGT: -25 ± 31.7* (*P* > 0.05)	Δ NAFLD fibrosis score: *P* > 0.05 Δ Liver stiffness: *P* < 0.001 Δ ALT: *P* > 0.05 Δ GGT: *P* > 0.05	5
						Control group	Δ NAFLD fibrosis score: -2.2 ± 1.1 (*P* > 0.05) Δ Liver stiffness: 0.2 ± 2.4 (*P* > 0.05) Δ ALT: 0 ± 18.3* (*P* > 0.05) Δ GGT: 9 ± 63.5* (*P* > 0.05)		
Mediterranean Diet (MD) vs. Low Fat Diet (LFD)									
Ristic-medic et al. 2020 [55]	Ultrasound	RCT, Parallel 3 months	*n* = 24 overweight/moderately obese patients with NAFLD (0% female) age 27–42 Serbia	FFQ	Energy-restricted diets for both groups (600–800 kcal/d reduction) 1. MD (50% CHO, 15% protein, 35% fat) 2. LFD (60% CHO, 15% protein, 25% fat)	MD	Δ FLI: -38.7 ± 6.0 (*P* < 0.001) Δ HIS: -8.3 ± 3.0 (*P* < 0.001) Δ ALT: -38 ± 19.1 (*P* < 0.001) Δ AST: -12.5 ± 10.5 (*P* < 0.001) Δ GGT: -23.1 ± 27.9 (*P* < 0.001)	Δ FLI: *P* = 0.02 Δ HIS: *P* = 0.43 Δ ALT: *P* = 0.13 Δ AST: *P* = 0.02 Δ GGT: *P* = 0.22	5
						LFD	Δ FLI: -28.4 ± 11.6 (*P* < 0.001) Δ HIS: -8.1 ± 2.7 (*P* < 0.001) Δ ALT: -31.2 ± 9.5 (*P* < 0.001) Δ AST: -10.5 ± 0.1 (*P* = 0.006) Δ GGT: -15.4 ± 6.5 (*P* < 0.001)		
Pintó et al. 2019 [53]	NMR	RCT, Parallel 3 years	*n* = 100 NAFLD patients with high cardiovascular risk (37% female) mean age: 64 ± 6 Spain	FFQ	1. MD + EVOO (60 mL of extra-virgin olive oil per day) 2. MD + mixed nuts (15 g walnuts, 7.5 g hazelnuts, and 7.5 g almonds per day) 3. LFD (recommended to reduce fat)	MD + EVOO	Δ Liver fat:1.2% [0–4.4] (*P* > 0.05) Δ ALT: 19.2 [16.6–28.4] (*P* > 0.05)	Δ Liver fat: *P* = 0.07 Δ ALT: *P* = 0.27	5
						MD + nuts	Δ Liver fat: 2.7% [0.2–11.0] (*P* > 0.05) Δ ALT: 26.0 [19.8–36.5] (*P* > 0.05)		
						LFD	Δ Liver fat: 4.1% [0.6–10.4] (*P* > 0.05) Δ ALT: 22.8 [17.1–38.1] (*P* > 0.05)		
Mediterranean Diet (MD) combined with low carbohydrate diets									
Properzi et al. 2018 [54]	MRS	RCT, Parallel 3 months	*n* = 48 patients with NAFLD (49% female) mean age: 51 ± 13.4 Australia	Daily self-assessed checklists	1. MD (40% CHO, 35–40% fat, 20% protein) 2. LFD (50% CHO, 30% fat, 20% protein)	MD	Δ hepatic fat: -10.2 ± 9.9 (*P <* 0.001) Δ ALT: -8 ± 31.2 (*P* = 0.049) Δ GGT: -19 ± 72.0 (*P* < 0.001)	Δ hepatic fat: *P* = 0.72 Δ ALT: *P* = 0.36 Δ GGT: *P* = 0.72	5
						LFD	Δ hepatic fat: -6.2 ± 6.0 (*P <* 0.001) Δ ALT: -12 ± 40.3 (*P* = 0.004) Δ GGT: -19 ± 75.6 (*P* = 0.05)		
Biolato et al. 2019 [44]	Liver biopsy	CCT, crossover 12 months	*n* = 20 patients with NAFLD (10% female) median age 43 Italy	24-h Dietary Recall and FFQ	Energy-restricted diets for both groups (1400 kcal/d) 1. MD (40% CHO, 20% protein, 40% fat) 2. LFD (62% CHO, 20% protein, 18% fat)	MD	Δ ALT: -28.3 ± 25 (*P* = 0.001) Δ AST: -6.4 ± 9.7 (*P* = 0.01) Δ GGT: -12.8 ± 7.0 (*P* > 0.05)	-	3
						LFD	Δ ALT: 2.1 ± 23.4 (*P* > 0.05) Δ AST: 2.5 ± 6.9 (*P* > 0.05) Δ GGT: -30.0 ± 81.0 (*P* > 0.05)		
Gepner et al. 2019 [47]	MRI	RCT, Parallel 18 months	*n* = 278 abdominal obesity/dyslipidemia patients with NAFLD (11% female) mean age: 48 Israel	FFQ	1. MD/LC (< 40 g/day CHO with a gradual increase up to 70 g/day, an increased protein and fat intake + 28 g walnuts) 2. LFD (30% fat with 10% saturated fat and 300 mg of cholesterol)	MD/LC	Δ Hepatic fat (%): -7.3 ± 9.2	Δ Hepatic fat (%): *P* = 0.04	5
						LFD	Δ Hepatic fat (%): -5.8 ± 7.2		
Ryan et al. 2013 [56]	biopsy-proven NAFLD/MRS	RCT, cross-over 18 weeks	*N* = 12 patients with NAFLD (50% female) mean age: 55 ± 14 Australia	Food records	1. MD (40% CHO, 20% protein, 40% fat) 2. LFD/HCD (50% CHO, 20% protein, 30% fat)	MD/LC	Δ IHL%: -5.6 ± 7.4 (*P* < 0.05) Δ ALT: -4.0 ± 17 (*P* > 0.05) Δ GGT: -4.0 ± 19.2 (*P* > 0.05)	Δ IHL%: *P* = 0.01 Δ ALT: *P* > 0.05 Δ GGT: *P* > 0.05	5
						LFD/HCD	Δ IHL%: -1.2 ± 2.6 (*P* > 0.05) Δ ALT: -4.0 ± 19.8 (*P* > 0.05) Δ GGT: -3.0 ± 17.4 (*P* > 0.05)		
Abbate et al. 2021 [41]	Ultrasound	RCT, Parallel 6 months	*n* = 128 patients with MetS and NAFLD (40%female) age 40–60 Spain	FFQ	Energy-restricted diets for all groups 1. LGIMD–high meal frequency (40–45% CHO (LGI), 25% protein, 30–35% fat, 7 meals a day) 2. LGIMD–PA (> 40–45% CHO (LGI), 20% proteins, 35–40% fat, 4–5 meals a day) 3. Conventional Diet (followed the U.S. Department of Agriculture recommendations with 45–65% CHO, 10–35% protein, 20–35% fat	LGIMD–high meal frequency	Δ Mean liver fat (%): -6.6 ± 10.6 (*P* < 0.001) Δ ALT: -3.2 ± 16.2 (*P* < 0.01) Δ AST: -14.5 ± 34.5 (*P* > 0.05) Δ GGT: -11.9 ± 71.7 (*P* > 0.05)	Δ Mean liver fat (%): *P* = 0.03 Δ ALT: *P* > 0.05 Δ AST: *P* > 0.05 Δ GGT: *P* > 0.05	5
						Conventional Diet	Δ Mean liver fat (%): -4.9 ± 7.8 (*P* < 0.001) Δ ALT: -4.4 ± 8.6 (*P* < 0.001) Δ AST: -11.4 ± 17.2 (*P* < 0.01) Δ GGT: -14.7 ± 23.2 (*P* < 0.001)		
Franco et al. 2020 [46]	FibroScan	RCT, Parallel 3 months	*n* = 144 patients with moderate or severe NAFLD (38% female) mean age: 49.9 ± 10 Italy	FFQ	1. LGIMD (no more than 10% of total daily calories are derived from saturated fats and an LGI with high MUFA and ω_3_PUFA) 2. Control Diet (based on Italy guidelines) 3. Aerobic Activity Program 4. Combined Activity Program 5. LGIMD + Aerobic Activity Program 6. LGIMD + Combined Activity Program	LGIMD	Δ NAFLD score: (-34.84, 95% CI -64.34; − 5.34) (*P =* 0.02)	Δ NAFLD score: *P* = 0.15	3
						Control Diet	Δ NAFLD score: (-24.46, 95% CI -56.07; 7.15) (*P =* 0.13)		
Misciagna et al. 2017 [51]	Ultrasound	RCT, Parallel 6 months	*n* = 98 moderate to severe NAFLD patients (26% female) age 30–79 Italy	Dietary record	1. LGIMD (50% CHO, 15–20% protein, 30% fat) 2. Control diet (healthy diet recommended by WHO)	LGIMD	Δ FLI: -23.5 ± 20.6 (*P <* 0.05)	Δ FLI: *P* < 0.05	5
						Control diet	Δ FLI: -9.6 ± 16.2 (*P <* 0.05)		
Marin-Alejandre et al. [49] 2019	MRI	RCT, Parallel 6 months	*n* = 98 patients with NAFLD and overweight or obese (47% female) age 40–80 Spain	FFQ	PA and energy-restricted diets for both groups (1200–1500 kcal/d) 1. FLiO (based on the MD principles with 40–45% CHO (LGI), 25% protein, 30–35% fat) 2. Control (followed by the American Heart Association with 50–55% CHO, 15% protein, 30% fat)	FLiO (LGIMD)	Δ Liver fat (%): -4.2 ± 3.5 (*P <* 0.001) Δ Liver stiffness: -0.1 ± 0.5 (*P* = 0.18) Δ FLI: -29 ± 14.6 (*P <* 0.001) Δ AST: -2 ± 5.3 (*P* = 0.30) Δ ALT: -11.6 ± 11.9 (*P <* 0.001) Δ GGT: -7.2 ± 27.5 (*P <* 0.001)	Δ Liver fat (%): *P* = 0.71 Δ Liver stiffness: *P* = 0.06 Δ FLI: *P* = 0.12 Δ AST: *P* = 0.12 Δ ALT: *P* = 0.47 Δ GGT: *P* = 0.69	3
						Control	Δ Liver fat (%): -3.6 ± 3.3 (*P <* 0.001) Δ Liver stiffness: 0.1 ± 0.5 (*P* = 0.20) Δ FLI: -26 ± 14.6 (*P <* 0.001) Δ AST: -3.9 ± 7.1 (*P <* 0.001) Δ ALT: -10.2 ± 11.2 (*P <* 0.001) Δ GGT: -12.6 ± 17.4 (*P <* 0.001)		
Low Carbohydrate Diet									
Browning et al. 2011 [45]	MRS	RCT, Parallel 2 weeks	*N* = 18 patients with NAFLD (72.8% female) mean age: 45 ± 12 USA	Dietary record	1. Carbohydrate-restricted diet (8% CHO, 33% protein, 59% fat) 2. Calorie-restricted diet (1500 kcal for men and 1200 kcal for women with 50% CHO, 16% protein, 34% fat)	Carbohydrate-restricted diet	Δ Hepatic fat (%): -55 ± 14 (*P* < 0.001) Δ AST: -33 ± 18 (*P* < 0.05) Δ ALT: 18 ± 47.6 (*P* > 0.05)	Δ Hepatic fat (%): *P* < 0.001 Δ AST: *P* = 0.30 Δ ALT: *P* = 0.80	3
						Calorie-restricted diet	Δ Hepatic fat (%): -28.0 ± 23.0 (*P* < 0.001) Δ AST: -41 ± 26.4 (*P* < 0.05) Δ ALT: 4 ± 30 (*P* > 0.05)		
Pe´rez-Guisado et al. 2011 [52]	Ultrasound	Single arm 12 weeks	*N* = 14 obese patients with MS and NAFLD (0% female) mean age: 41.18 ± 2.28 Spain	Not specified	1. SKMD (unlimited energy diet with < 30 g/day CHO)	SKMD	Δ ALT: -34.8 ± 9.2 (*P* < 0.001) Δ AST: -18.4 ± 7.6 (*P* < 0.001)	-	3

^1^Values are means ± SDs or [IQR] unless otherwise indicated. *Medians ± SDs

ALT: alanine transaminase; AST: aspartate transaminase; ALP: alkaline phosphatase; BLS: bright liver score; CCT: controlled clinical trial; EVOO: extra-virgin olive oil; FLI: fatty liver index; FLiO: fatty liver in obesity; GGT: gamma-glutamyl transferase; HDG: healthy dietary guidelines; HFC: hepatic fat content; HIS: hepatic steatosis index; IHF: intrahepatic fat; LFD: low-fat diet; LF/HCD: low fat/high carbohydrate diet; LGIMD: low glycemic index Mediterranean diet; MD: Mediterranean diet; MD/LC: Mediterranean/low-carbohydrate; MLG: Mediterranean lifestyle group; MetS: metabolic syndrome; MRI: magnetic resonance imaging; MRS: magnetic resonance spectroscopy; MUFA: monounsaturated fatty acids; NMR: nuclear magnetic resonance; PA: physical activity; PUFA: polyunsaturated fatty acids; RCT: randomized controlled trial; SKMD: Spanish ketogenic Mediterranean diet

### Observational studies

Table 2 shows the characteristics of observational studies investigating the association of dietary patterns with nonalcoholic fatty liver disease, with 15 studies using a priori dietary patterns as follows:

#### Mediterranean dietary scores (MDS)

Of the 11 studies that assessed adherence to the MDS [5, 16–18, 23, 28–33], six reported that the MDS was inversely associated with steatosis or fibrosis, including two cohort studies [31, 32], one case-control study [23], and three cross-sectional studies [5, 17, 28]. The cohort study in the US used CT scans in the second-and third-generation Framingham Heart Study cohorts [32], and the cohort study in Greece used the TyG index [31].

Some articles reported nonsignificant results for adherence to the MDS: The Multiethnic Cohort Study (MEC) in the US using linkage to 1999–2016 Medicare claims [33], the Rotterdam study in the Netherlands using transient elastography [16], and the Swiss CoLaus Study in Switzerland using the FLI and NAFLD score for NAFLD diagnosis [29].

#### Dietary approach to stop hypertension (DASH)

In the four studies that reported adherence to the DASH diet, the MEC study [33], and two cross-sectional studies using MRI and ultrasound for diagnosis [5, 38], a significant inverse association between adherence to the DASH diet and NAFLD was reported. A case-control study by Hekmatdoost et al. using a fibroscan [25] showed null results.

#### Healthy eating index (HEI) and alternative healthy eating index (AHEI)

In the MEC study, adherence to HEIs was significantly associated, while adherence to AHEIs was not associated with a reduced risk of NAFLD [33]. In the Framingham cohort study, adherence to the AHEI was associated with a 21% decrease in the incidence of fatty liver [32]. A case-control study in Iran using ultrasound for diagnosis revealed no association between adherence to HEIs and NAFLD [24].

#### Other *priori* dietary patterns

A few studies evaluated adherence to the WHO score [16], Dutch dietary guidelines [16], dietary diversity score [24], Diet Quality Index International [18], and a vegetarian diet [19] (Table 2).

As Table 2 shows, 12 studies used *posteriori* dietary patterns. All of the *a posteriori* dietary patterns were extracted from case-control or cross-sectional studies. Studies that used *a Posteriori* dietary method showed that unhealthy dietary patterns [40], such as Western dietary patterns [34, 35], high carbohydrate intake, snacks, and sweets [15, 26, 36, 37], animal foods [37, 39], fast food [21, 22, 27, 37], and high meat [22, 36], were positively associated with NAFLD, whereas adherence to healthy dietary patterns [15, 34], plant-based food patterns [15, 35, 39, 40], dietary patterns consisting of high amounts of vegetables, low-fat dairy [21, 36], legumes [36], and simple meal patterns [20] were associated with a reduced risk of NAFLD. Nonsignificant associations were reported between some *a posteriori-driven* dietary patterns and NAFLD [15, 20, 21, 26, 27, 37], such as a high-protein diet [20, 27]. The traditional dietary patterns (TDPs) were country-specific, and the associations with NAFLD were not consistent. Negative [21, 22], positive [15, 20, 39], and null associations [34, 37] were reported for various *a posteriori* dietary patterns.

The limited number of studies precludes the conduct of a meta-analysis.

### Interventional studies

We report the results of interventional studies for two outcomes: changes in NAFLD severity indices and changes in the levels of various liver enzymes.

#### NAFLD severity indices

All 16 articles reported a significant improvement in hepatic fat accumulation or stiffness despite intervention with a variety of dietary indices used as follows:

##### Mediterranean Diet (MD)

*MD vs. control*: All four studies showed that adherence to an MD is more effective than a habitual or healthy diet [42, 43, 48, 50].

##### Low-fat diet (LFD)

Of five studies [47, 53–56] investigating the effect of low-fat diets on liver steatosis, three found a significant reduction in liver steatosis.

##### *MD vs. LFD*

Two studies have investigated the effect of MD vs. LFD on liver steatosis. One study on 24 obese patients showed that an MD is more beneficial than a low-fat diet [55], and another study on 100 patients with high cardiovascular risk showed no significant difference between these two dietary patterns [53].

##### *MD combined with low carbohydrate diets vs. LFD or control*

Other studies have reported the effect of low carbohydrate plus MD (MD/LC) or low glycemic index plus MD (LGIMD) or carbohydrate-restricted diet vs. LFD or control group (following a healthy dietary pattern) on NAFLD [41, 45–47, 49, 51, 54, 56]. In summary, following a low carbohydrate/low glycemic index diet is effective in reversing steatosis.

##### DASH

Other dietary patterns, including DASH, have been investigated in a few studies. The only RCT evaluating the DASH diet was excluded as it had been flagged with an expression of concern by the publishers [14].

##### Low-Carbohydrate Diet

While there is no scientific consensus on the exact level of carbohydrate intake that defines a low-carbohydrate diet [58], some studies have utilized it as an intervention. According to the Browning et al. [45] study, two weeks of a low-carbohydrate diet (8% carbohydrate, 33% protein, 59% fat) significantly improved hepatic fat in 18 patients with NAFLD.

##### Liver enzymes

A total of 12 studies measured changes in alanine transaminase (ALT) [41–45, 48, 49, 52–56], five studies measured changes in aspartate transaminase (AST) [41, 45, 49, 52, 55], and nine studies measured changes in gamma-glutamyl transpeptidase (GGT) [41–44, 48, 49, 54–56].

##### MD

*MD or LGIMD vs. control*. In five studies that investigated the effect of MD or LGIMD vs. conventional diets on liver enzymes, no difference was found between the groups [41–43, 48, 49].

##### LFD

Two studies showed that a low-fat diet may improve liver enzymes [54, 55], and three studies showed null results [41, 51, 55].

*MD vs. LFD or LFD/High Carbohydrate Diet (LFD/HCD)*:

In nine studies investigating the effect of MD vs. LFD/HCD on transaminase enzymes, no difference was observed between the two groups [44, 47, 53–56]. Only Ristic-medic et al. [55] and Biolato et al. [44] reported that AST and ALT significantly decreased in the MD and LFD groups, respectively (*P* = 0.02 and 0.04, respectively).

##### Low Carbohydrate Diet (LCD)

In a study by Browning et al. (20 g carbohydrate/day) [45], only AST decreased in both the carbohydrate- and calorie-restricted groups (*P* < 0.05). According to the Pe´rez-Guisado et al. [52] study, a 12-week Spanish Ketogenic Mediterranean Diet (SKMD) (< 30 g carbohydrate/day) significantly improved ALT (*P* < 0.001) and AST (*P* < 0.001) levels in 14 obese men with NAFLD.

### Sensitivity analyses

When we restricted our study to cohort studies to limit recall bias, two studies reported MD [29, 31], one study reported HEI and DASH [33], one study reported AHEI [32], and one study reported that WHO dietary patterns [16] are associated with a decreased risk of NAFLD. When we restricted our results to articles that used biopsy, MRI, or MRS as accurate methods for NAFLD diagnosis, we found that the MD, DASH, MD/LC, low-carbohydrate diet, and LFD methods are all beneficial for managing NAFLD [5, 18, 30, 44, 45, 47, 49, 50, 54, 56].

Several studies have evaluated the effects of supplements [42, 43], sleep habits [48], and physical activity [41, 48] as a separate group of interventions. We have not included these results in the tables since the purpose of this study was to evaluate dietary patterns only, and there were few of these separate groups.

## Discussion

Our study showed that most of the studies on dietary patterns and NAFLD evaluated the MD, and the evidence demonstrated that adhering to an MD is associated with a lower risk of NAFLD [5, 17, 23, 28, 31, 32]. Additionally, most interventional studies have shown the benefits of MD in improving intrahepatic lipids, which can ultimately improve NAFLD [41–44, 47–50, 54–56]. Additionally, the use of other healthy dietary patterns, such as DASH, or the manipulation of macronutrient distribution, such as a low-glycemia/low-carbohydrate diet, could be effective in reversing steatosis. Studies that used *a posteriori* diet showed that unhealthy dietary patterns, such as Western dietary patterns characterized by high consumption of sweets, red meat, and fast food, were positively associated with NAFLD.

### MD

Concerning the prevention of chronic disorders, one of the most well-known dietary patterns in the literature is the MD [59]. However, there are various MD scoring systems, such as 9-point and 14-point scales which could potentially challenge the validity of our results. The MD is characterized by an abundance of plant foods, vegetables, fruits, legumes, whole grains, nuts, fish, extra virgin olive oil, and less consumption of dairy, poultry, and red and processed meat [60]. Moreover, MD improves NAFLD through its antioxidant and anti-inflammatory effects. The MD is rich in monounsaturated fatty acids (MUFAs), omega-3 polyunsaturated fatty acids (PUFAs), fibers, and polyphenols, which have been found to have a beneficial effect on glycemic and lipoprotein metabolism and therefore on NAFLD. MUFAs improve metabolic parameters such as glycemic disorders, lipid metabolism, and blood pressure, decreasing the risk of NAFLD [61]. Omega-3 PUFAs show beneficial effects via a reduction in the inflammatory response and oxidative stress and improvement of insulin sensitivity and therefore can decrease hepatic steatosis [62]. Furthermore, dietary fiber can reduce the risk of type 2 diabetes, dyslipidemia, and NAFLD by increasing the production of short-chain fatty acids and phenolic compounds, both of which act as antioxidants and modulate the gut microbiota [63]. Polyphenols, as an important component of an MD, are found in foods such as olive oil, nuts, and red wine (when consumed in moderation). Mitochondrial dysfunction is directly associated with chronic diseases, and polyphenols exert their beneficial effects by regulating genes and signaling pathways that influence inflammation, mitochondrial function, and oxidative stress [64].

### DASH

The DASH diet pattern was associated with improved NAFLD parameters such as liver steatosis in observational studies [5, 25, 33, 38, 57]. However, its effectiveness in NAFLD management has rarely been investigated in interventional studies. Dietary patterns based on DASH emphasize the consumption of whole grains, legumes, seeds, nuts, vegetables, fruits, low-fat dairy products, fish, and chicken, with a reduction in red meat, fat, sweet, and sugary drink consumption [65]. Whole grains and nuts are important components of the DASH diet and contribute to the reduction of risk factors and disease severity of NAFLD due to the nutrients, fibers, and phytochemical composition [66, 67]. DASH diets also include fruits and vegetables with high natural antioxidants, which are beneficial for managing NAFLD [68]. The MD and DASH dietary patterns have most of their recommendations in common, but the MD does not include dairy, while the DASH contains low-fat dairy.

### Other dietary patterns

Furthermore, other studies have shown an inverse association between NAFLD and other healthy dietary patterns. Various labels were assigned to different healthy dietary patterns, including MD, DASH, HEI, AHEI, and plant-based diets. However, all nutrient-dense diets, that provide a high amount of essential nutrients, were associated with a reduced risk of NAFLD [5, 16, 18, 19, 32, 33, 38, 57]. This consistency across nutrient-dense dietary patterns with different names has been reported in other studies with different outcomes [69–72].

According to studies that assessed dietary patterns using the *a posteriori* method, unhealthy dietary patterns, including the Western dietary pattern, and dietary patterns characterized by high consumption of sweets and animal foods such as red meat and fast food were positively associated with NAFLD [21, 22, 27, 36, 37]. The Western dietary pattern is often regarded as a diet containing a high amount of refined sugars and saturated fats that can affect metabolic pathways and increase intrahepatic triglycerides by stimulating adipose tissue lipolysis. Furthermore, excess energy from simple sugars leads to an increase in triglycerides in the liver through de novo lipogenesis [73]. Previous studies evaluating the association between animal foods and sweets as a food item and NAFLD showed similar findings [74, 75].

In contrast, adherence to a healthy diet rich in high amounts of fruit, vegetables, legumes, and low-fat dairy was associated with a reduced risk of NAFLD [21, 36]. These findings are similar to those of previous studies on NAFLD and specific food items, including whole grains [76], legumes [77], low-fat dairy [78], and vegetables [75]. However, we observed conflicting results for fruits. In the Fakhoury-Sayegh et al. [22] study, a dietary pattern characterized by more than 2–3 servings/d of fruits increased the odds of NAFLD, while Tutunchi et al. [36] reported that a healthy dietary pattern characterized by high consumption of fruit, vegetables, legumes, and low-fat dairy products was associated with a reduced risk for NAFLD. Furthermore, in healthy dietary patterns, approximately 2 servings/day of fruits are recommended. This discrepancy may be explained by the fact that fruits contain simple carbohydrates, such as fructose, which has been associated with NAFLD and metabolic syndrome components, including visceral adiposity, dyslipidemia, insulin resistance, and hypertension [79]. When exposed to high loads of fructose, enterocytes metabolize more fructose locally, leading to an increase in de novo lipogenesis. Excess fructose can contribute to the formation of triglycerides, which may enter circulation, raising the risk of developing NAFLD or other metabolic disorders [80]. The discrepancy observed may also stem from limitations inherent in certain methodologies used for dietary data collection.

### Diet based on macronutrient distribution

The optimal distribution of macronutrients to improve NAFLD is unclear. However, the current review revealed that manipulating the macronutrient composition by restricting carbohydrate content [41, 45–47, 49, 51, 56] plays a key role in improving NAFLD. Nevertheless, there is still no scientific consensus on the specific level of carbohydrate intake that defines a low-carbohydrate diet [58]. A few studies have shown that a low-fat diet may improve NAFLD [47, 54, 55]. An LFD usually limits fat energy to less than 30% of total daily calories, while less than 20% is considered a very LFD [81]. However, we noticed that the authors defined the low-fat diet differently, from 18 to 30%, or even only advised the participants to decrease fat intake [53]. This could be the reason for inconsistent results on the effect of LFD on NAFLD. More studies are needed to compare LFD with MD and LFD with LCD/LGI.

### Strengths and limitations

Providing up-to-date information about NAFLD and different dietary patterns and manipulating macronutrient distribution using both observational and interventional studies is one of the main strengths of this systematic review. We used two popular methods to evaluate the risk of bias in the studies, and most studies had a low to moderate risk of bias. Moreover, it encompasses a variety of populations with different dietary patterns and various designs, providing a more reliable and generalized view of the topic from various perspectives. However, there are several limitations. The first limitation is that there is heterogeneity in studies regarding the duration of intervention and follow-up, characteristics of participants, the timing of data collection, and outcome criteria. Different methods have been used for NAFLD diagnosis, and even studies that used an accurate method for diagnosis, such as biopsy, did not repeat the accurate method for follow-up. Varying diagnostic criteria can result in misclassification bias. Studies that use imaging techniques (e.g., ultrasound, MRI) may have different sensitivity and specificity compared to studies using liver biopsy. This makes it difficult to directly compare results across studies and reduces the strength of any pooled estimates. Variations in dietary assessment tools like FFQs, 24-hour recalls, or dietary diaries with distinct advantages and limitations can introduce bias or variability in the results. Differentiating between the scoring systems of dietary patterns such as MD in the analysis was beyond the scope of this study. The transition from NAFLD to MAFLD toward the end of the study period limited the available studies on MAFLD, highlighting the need for future systematic reviews [82]. Moreover, publication bias should be considered in this systematic review. All published studies were analyzed, while unpublished studies were not considered. The dietary patterns extracted via exploratory methods may vary in different populations; for example, the Western dietary pattern in one population is not the same as that in another population. Since this review takes a comprehensive approach, the methodological heterogeneity of the dietary patterns covered allows for qualitative analysis.

### Recommendations for future studies

The aforementioned limitations prompted us to develop reporting recommendations for future studies to fill the gap in knowledge regarding the management of NAFLD via dietary modifications:

- More research in different countries is needed. There were no studies from Africa or South America.
- The effectiveness of the DASH diet for NAFLD management has rarely been investigated in interventional studies.
- All of the *a posteriori* dietary patterns were extracted from cross-sectional or case-control studies that are prone to recall bias. The use of cohort studies is recommended for more exploratory dietary patterns.
- More interventional research is needed to evaluate the optimal macronutrient proportions. More research on low-fat diets, especially on the percentage of fat as well as the type of fats, including MUFAs and PUFAs, is needed. More studies are needed to compare LFD with MD and LFD with LCD/LGI.
- More interventional studies on the effect of fruit consumption on NAFLD are needed.
- More high-quality research using accurate methods for the diagnosis of NAFLD in both diagnosis and follow-up is needed.
- Given the recent shift in terminology from NAFLD to MAFLD, future studies would benefit from utilizing the updated MAFLD criteria in literature searches and analysis.

## Conclusions

Our results demonstrated that following a healthy dietary pattern such as the MD or DASH diet is an effective way to prevent and manage NAFLD. All other healthy dietary patterns characterized by high consumption of vegetables, whole grains, nuts and legumes, vegetable oils, and fish were associated with a lower risk of NAFLD, and unhealthy dietary patterns such as Western dietary patterns characterized by high consumption of sweets, red meat, and fast food were positively associated with NAFLD. A low-carbohydrate diet is effective at preventing and treating NAFLD; however, more research on a low-fat diet is needed.

## Data Availability

No datasets were generated or analysed during the current study.

## Abbreviations

AHEI: Alternative healthy eating index
ALP: Alkaline phosphatase
ALT: Alanine transaminase
AST: Aspartate aminotransferase
BMI: Body mass index
DASH: Dietary approaches to stop hypertension
HEI: Healthy Eating Index
EVOO: Extra virgin olive oil
FLI: Fatty liver index
LCD: Low-carbohydrate diet
LFD: Low-fat diet
LF/HCD: Low fat/high carbohydrate diet
LGIMD: Low glycemic index Mediterranean diet
MD: Mediterranean diet
MD/LC: Mediterranean/low-carbohydrate
MRI: Magnetic resonance imaging
MRS: Magnetic resonance spectroscopy
MUFA: Mono-unsaturated fatty acid
NAFLD: Nonalcoholic fatty liver disease
NASH: Nonalcoholic steatohepatitis
NMR: Nuclear magnetic resonance
PUFA: Polyunsaturated fatty acid
SKMD: Spanish Ketogenic Mediterranean Diet
TDP: Traditional dietary patterns
TyG: Triglycerides-glucose
WDP: Western dietary patterns
